# Video augmentation of the WHO cone assay to quantify mosquito behavioural responses to insecticide-treated nets

**DOI:** 10.1186/s13071-023-06029-z

**Published:** 2023-11-15

**Authors:** Jeff Jones, Agnes Matope, Priscille Barreaux, Katherine Gleave, Keith Steen, Hilary Ranson, Philip J. McCall, Geraldine M. Foster

**Affiliations:** Department of Vector Biology Liverpool School of Tropical Medicine, Pembroke Place, Liverpool, UK

**Keywords:** Vector control, Behaviour, *Anopheles gambiae* s.l., Insecticide-treated net, Malaria, Mosquito, Assay, Testing, WHO cone test, Insecticide resistance

## Abstract

**Background:**

Insecticide-treated nets (ITNs) using pyrethroids have been the main vector control tools deployed in malaria endemic countries and are responsible for the dramatic reduction in African malaria cases in the early 2000s. The World Health Organization (WHO) cone test was designed to assess the rapid toxicity effects of pyrethroid exposure on mosquito vectors but has yielded no insights beyond 60-min knockdown and 24-h mortality. As dual-active-ingredient (AI) ITNs become more widespread, bioassays that can provide realistic assessment of single- and dual-treated ITNs (i.e. nets with more than one active ingredient) are urgently needed.

**Methods:**

We present an augmentation of the cone test that enables accurate quantification of vector behavioural responses (specifically movement, spatial and temporal occupancy) to ITNs using video recording and bespoke software that uses background segmentation methods to detect spatial changes in the movement of mosquitoes within the cone. Four strains of *Anopheles gambiae* sensu lato (s.l.) were exposed to four ITNs (PermaNet 2.0, PermaNet 3.0, Olyset Net, Interceptor G2) and untreated nets in these modified cone tests. Life history data (post-exposure blood-feeding, blood meal weight, longevity) for individual mosquitoes were recorded.

**Results:**

All mosquitoes responded to the presence of ITNs, spending from 1.48 to 3.67 times more time in the upper region of the cone, depending on the ITN type. Of all ITNs, PermaNet 2.0 provoked the smallest change in behavioural response. Activity in the cone influenced observed post-exposure longevity, and in resistant strains exposed to Interceptor G2, the higher the activity, the greater the risk of dying, as long as the proportion of activity at the net surface was less than 50%. All ITNs inhibited blood-feeding, and smaller blood meals were taken when mosquitoes fed.

**Conclusions:**

The additional mosquito behaviour data obtained by using this modification to the WHO cone test provides unique insight into the innate responses of different mosquito strains on untreated nets and the entomological mode of action of ITNs, important evidence when evaluating ITN characteristics.

**Graphical Abstract:**

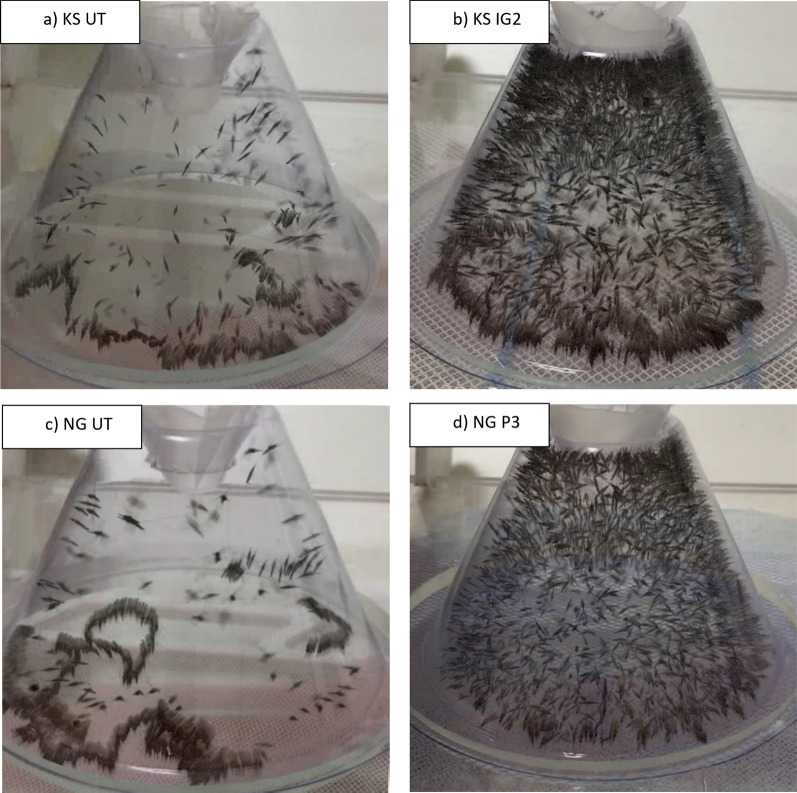

**Supplementary Information:**

The online version contains supplementary material available at 10.1186/s13071-023-06029-z.

## Background

Insecticide-treated nets (ITNs) have contributed substantially to a decline in the burden of malaria caused by the principal African malaria vectors *Anopheles gambiae* sensu lato (s.l.) and *Anopheles funestus* in at-risk communities across Africa [1, 2]. Seemingly simple tools, ITNs achieve an impact through a multifunctional mechanism that reduces the contact rate between humans and malaria vectors, alters blood-feeding and host-seeking behaviours, and causes mosquito death [3–7]. Increased insecticide resistance has reduced the instantaneous killing capacity of ITNs, but the effects of ITNs on host-seeking behaviours and blood-feeding remain, regardless of the insecticide resistance status of local vector populations [8–14].

Assessments of ITN impact are principally conducted using the World Health Organization (WHO) cone test and tunnel tests during laboratory testing and experimental hut trials during semi-field testing [15]. The most widely used laboratory evaluation method is the WHO cone test, where five mosquitoes are exposed to a net surface for 3 min, and knockdown at 60 min and mortality at 24 h are collected as parameters by which to adjudge ITN efficacy [15]. The test is designed to capture the rapid toxicity effects induced by fast-acting pyrethroid insecticides, and yields no insight into mosquito behaviours that might influence ITN performance [16] or the effects of compounds that target different stages of the mosquito life cycle or which induce delayed mortality [17–20]. With multiple classes of insecticides now in use as ITN treatments, there is an urgent need for ITN evaluation methods to accurately measure a broad range of insecticidal effects [21].

Recognising the range of immediate and delayed impacts of ITNs requires a thorough understanding of the effects of insecticide exposure. For example, if a mosquito successfully takes a blood meal through an ITN, its chances of survival are higher than a mosquito that is exposed but remains unfed [22]. ITNs reduce overall post-exposure (PE) blood-feeding success, blood-feeding duration and the time spent on the net, relative to the untreated (UT) net [23]. Pyrethroid-resistant mosquitoes that are not immediately killed by an ITN may nonetheless exhibit a reduced life span [24], and the effects of pro-insecticides that require metabolization into active forms may be enhanced by assays that allow mosquitoes to have a free range of movement [25].

Advances in video recording and machine vision techniques have enabled fine-grained studies of mosquito behaviours ranging in scale from house entry dynamics [26] to flight distribution within a small wind tunnel [27]. Measuring key behavioural parameters during and after ITN exposure provides additional insights about the likely effect of a given ITN on a particular vector population. In an experimental field hut study, mosquitoes reduced the proportion of time spent in a ‘bouncing’ flight mode when an ITN was present [28]. Tracking of mosquito positions in smaller-scale assays can quantify net contact and excito-repellency effects, and investigate the relationship between these parameters and observed mortality [14].

To address this goal, we present an automated method, video cone test analysis (ViCTA), designed to use the video output of mosquito activity inside the cone recorded during the video-modified WHO cone assay, as recently reported by Hughes et al. [14], where mosquito behavioural states were manually assessed at 5-s intervals. In this new study, video recordings of standard WHO cone assays are automatically analysed using background subtraction methods to detect and segment moving mosquitoes at 0.1-s intervals. The detected movements are aggregated to report total movement activity and total and proportional distribution of mosquitoes within the cone, before screening for correlation with a range of subsequent life history outcomes to evaluate for indications of the ‘whole life’ impact of ITNs on mosquitoes (Additional file 1: Fig. S1).

## Methods

### Physical apparatus

**Fig. 1:**
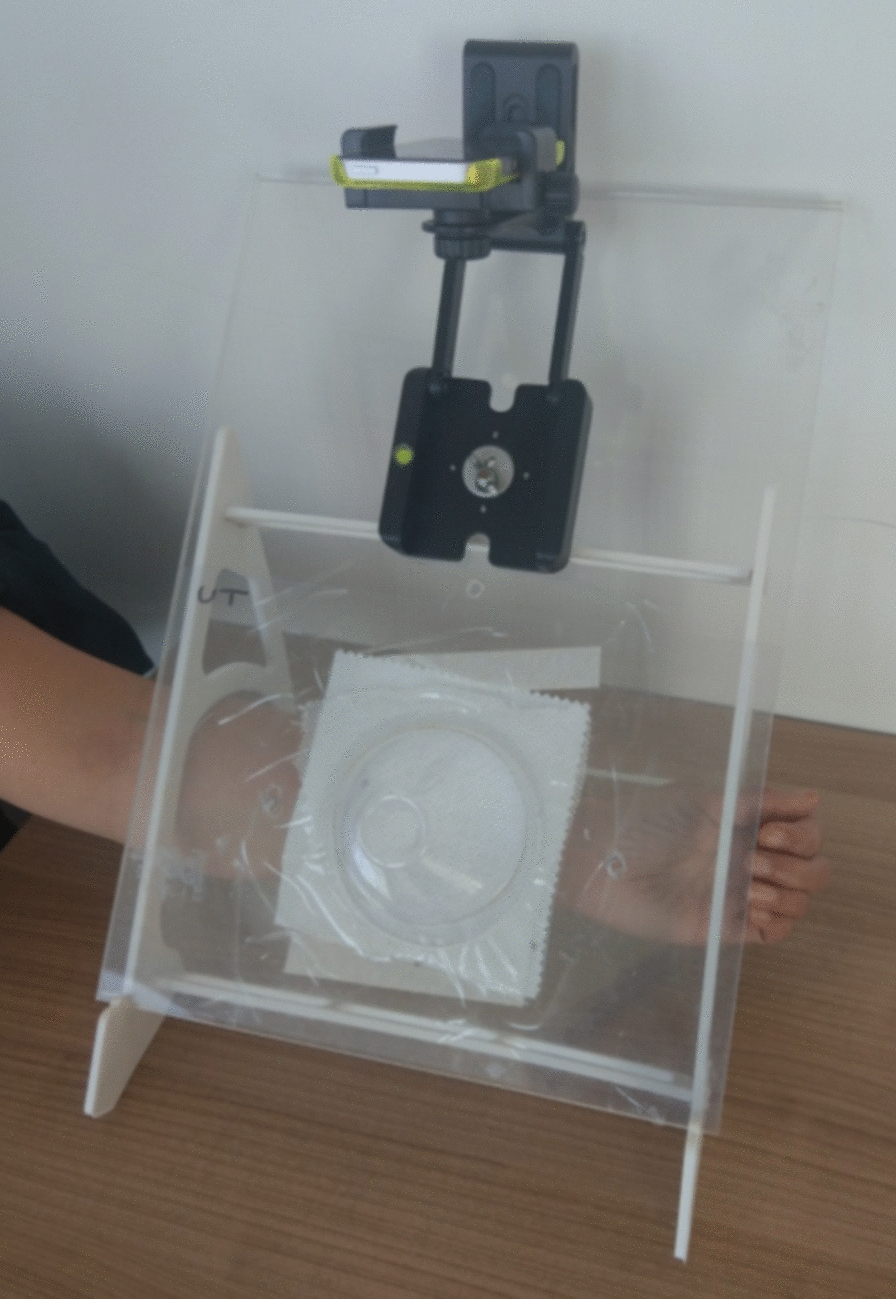
Experimental rig for video cone test analysis. The test netting is sandwiched between two pieces of 3-mm acrylic mounted at 45° and containing a 9-cm hole to allow exposure to the host stimulus scent. The host rests its arm below the scent stimulus hole, and mosquitoes are not able to contact the host arm. A smartphone is placed above the cone and immobilised at a fixed distance and angle using a z-clamp

The WHO cone and smartphone (Apple iPhone SE, Apple Inc., Cupertino, CA, USA) were mounted on a bespoke frame as per Hughes et al. [14], shown in Fig. 1.

### Insecticidal netting

A UT net (Bayer, Germany) was used in control tests. ITNs tested were the PermaNet 2.0 (Vestergaard Frandsen, Switzerland, deltamethrin 1.4/1.8 g/kg, P2), PermaNet 3.0 [Vestergaard Frandsen, Switzerland, deltamethrin 4.0 g/kg (roof), 2.8/1 g/kg (sides), piperonyl butoxide (PBO) 4.0 g/kg (roof), P3], Olyset Net [Sumitomo Chemical Co., Ltd., Japan, 2% (w/w) permethrin 20 g/kg, OS], and Interceptor G2 (BASF, Germany, alpha-cypermethrin 100 mg/m^2^, chlorfenapyr 200 mg/m^2^, IG2). ITNs were aired at ambient temperature for 1 week, then stored at 4 °C until acclimatisation at laboratory temperature 24 h prior to testing.

### Mosquito strains

Experiments were performed at the Liverpool School of Tropical Medicine (LSTM) using four colonised strains: the insecticide-susceptible Kisumu (KS) (*An. gambiae* sensu stricto) and N’gousso (NG) (*Anopheles coluzzii)* strains and pyrethroid-resistant Banfora (BF) and VK7 strains (both *An. coluzzii).* Both insecticide-resistant strains are maintained under 6-monthly selection pressure with deltamethrin and have been previously described [29, 30].

All experiments were performed in a climate-controlled laboratory (27 ± 2 °C, 80 ± 8% RH) using non-blood-fed 3–5-day-old female mosquitoes. Mosquitoes were sugar-starved for at least 5 h prior to testing.

### ViCTA video cone tests

A mean of 43 cone test replicates each were performed for KS, NG and VK7 strains, respectively; fewer BF replicates (mean = 19) were conducted due to colony issues. All cone tests were conducted using the modifications to the WHO guidelines described in Hughes et al. with a host attractant. Mortality at 24 h and life history data were recorded.

### Life history data

The life history data collected were blood-feeding at 1 and 24 h PE, blood meal weight (BMW) and longevity as described in Hughes et al. A human arm was offered as a blood meal source at 1 h PE; mosquitoes that did not feed at 1 h were offered a second opportunity to blood-feed at 24 h PE. Mosquitoes were monitored individually in individual 50-ml Falcon tubes with a lid of UT netting. Seventy-two hours after blood-feeding, individual BMWs were measured using excreted haematin [31]. One millilitre of 1% lithium carbonate was used to dissolve all excreted haematin and the absorbance at 397 nm was measured in triplicate. A standard curve comprising standards from 1.76 to 30 µg/ml was used to calculate the total weight of haematin (µg/ml) in each sample. Mosquitoes were maintained in individual housing with access to 10% sugar solution until death. Mortality was recorded every 24 h. Wing length was measured after death and was used as an index of mosquito mass [32].

### Video recording and analysis

Video files were converted to 540 × 960 pixels, 30 frames per second, H264 encoding at 1500 Kb bitrate, inside an MPEG-4 container prior to analysis. Background segmentation methods detect actively moving mosquitoes within the cone and log the location of moving mosquitoes at 0.1-s intervals. Preprocessing morphological operators are used for each video frame and interpreted by a Gaussian mixture model for background segmentation [33] to generate regularised contour objects representing moving mosquitoes whilst removing noise artefacts (Supplementary methods 1).

Mosquito movement counts are calculated by the software and aggregated at 5-s intervals for 35 epochs of spatio-temporal activity per test. The first 5 s of the test is excluded to allow for acclimatisation of the background segmentation model. Metrics produced for data analysis include (i) total mosquito activity (movements of all five mosquitoes in the cone over 180 s) and (ii) regional activity (movements of all five mosquitoes in the cone stratified into the upper half (UHC) and lower half (LHC) of the cone volume. Frames with no moving mosquitoes constitute mosquito inactivity. Output result files were parsed and combined with post-test monitoring data using R.

### Statistical analysis

Statistical analysis was performed using R statistical software version 4.1.2 and R Studio [34, 35]. Descriptive statistics were generated using the number of observations, mean and standard deviation (SD) for continuous variables, the number and percentage of observations for categorical variables, and the number and percentage of observations, mean, SD, minimum, maximum, median survival and 95% confidence interval (CI) for time-to-event variables.

Behavioural data were analysed using linear mixed models (LMMs) and generalised linear mixed models (GLMMs) in the R lme4 and glmmTMB packages, respectively [36, 37]. The normal, negative binomial, and beta-binomial distributions were used to evaluate the total, regional and proportion of activity in the LHC, respectively. The mean difference, incidence risk ratio (IRR), odds ratio (OR) and 95% CIs were generated. Mosquito strain, treatment, number of inactive frames, valid frames proportion, and two-way interaction (treatment and strain) were included for all the models, and three-way interaction (treatment, strain, and region) and two-way interactions (region and inactive frames, and strain and region) for the regional activity model only. The test date and the operator within the test date were fitted as random effects for the regional and proportion of activity at LHC outcomes, respectively. The test date was too variable for total activity (model without test date fitted the data better) and hence was not retained in the final model.

For the life history data, GLMMs were employed to analyse PE blood-feeding success and 24-h mortality using the binomial distribution, and LMMs for the BMW, using the normal distribution, respectively. Longevity was analysed using the mixed-effects Cox regression model using the R ‘coxme’ package [38]. Treatment, wing-length measurement (BMV and longevity only), PE feeding time, BMW, the predicted proportion of activity in LHC (PLHC), two-way interactions (treatment and strain, treatment and PLHC, treatment and wing-length measurement) and three-interaction (strain, treatment and PE feeding time) for the PE blood-feeding success model only were included as fixed effects while replicate and test date were included as random effects.

The likelihood ratio test was used under the restricted maximum likelihood estimation to determine the statistical significance of the random effects. The fixed effects for the ‘best’ model were determined using the Akaike information criterion and the residuals based on the DHARMa package [39]. All comparisons were conducted within a strain comparing each ITN to UT using the ‘emmeans’ package [40]. Dunnett’s method was employed to control for the probability of making false-positive findings. Statistical significance was set at 5%.

### Ethical approval

Ethical approval for this laboratory study was obtained from the LSTM Research Ethics Committee as part of the project: Developing entomological indicators to assess the public health value of next-generation LLINs (protocol number 19-038, approval 06.08.2019). Volunteers acting as arm feeders were registered with LSTM as mosquito colony arm feeders and had previously signed consent forms which were kept on file.

## Results

### Total mosquito activity at untreated and treated nets

**Fig. 2:**
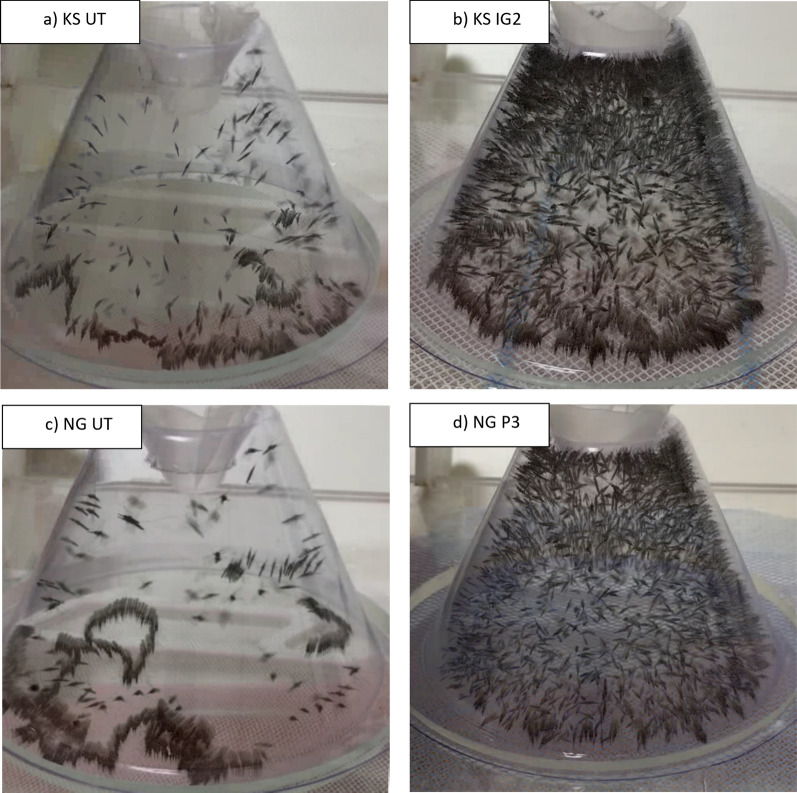
Composite outputs from individual ViCTA analyses demonstrate behavioural differences between untreated and insecticide-treated bednets. **a** composite *summary* image using the *Kisumu* strain of *An. gambiae* (KS) on an untreated net constructed by sampling video every 0.1 s and merging darkest components of each frame,** b** Composite *summary* output of KS on net treated with Interceptor G2 (IG2),** c** composite image of *N’gousso* strain of *An. coluzzii* (NG) on untreated net,** d** Composite summary output of NG exposed to PermaNet 3

**Fig. 3:**
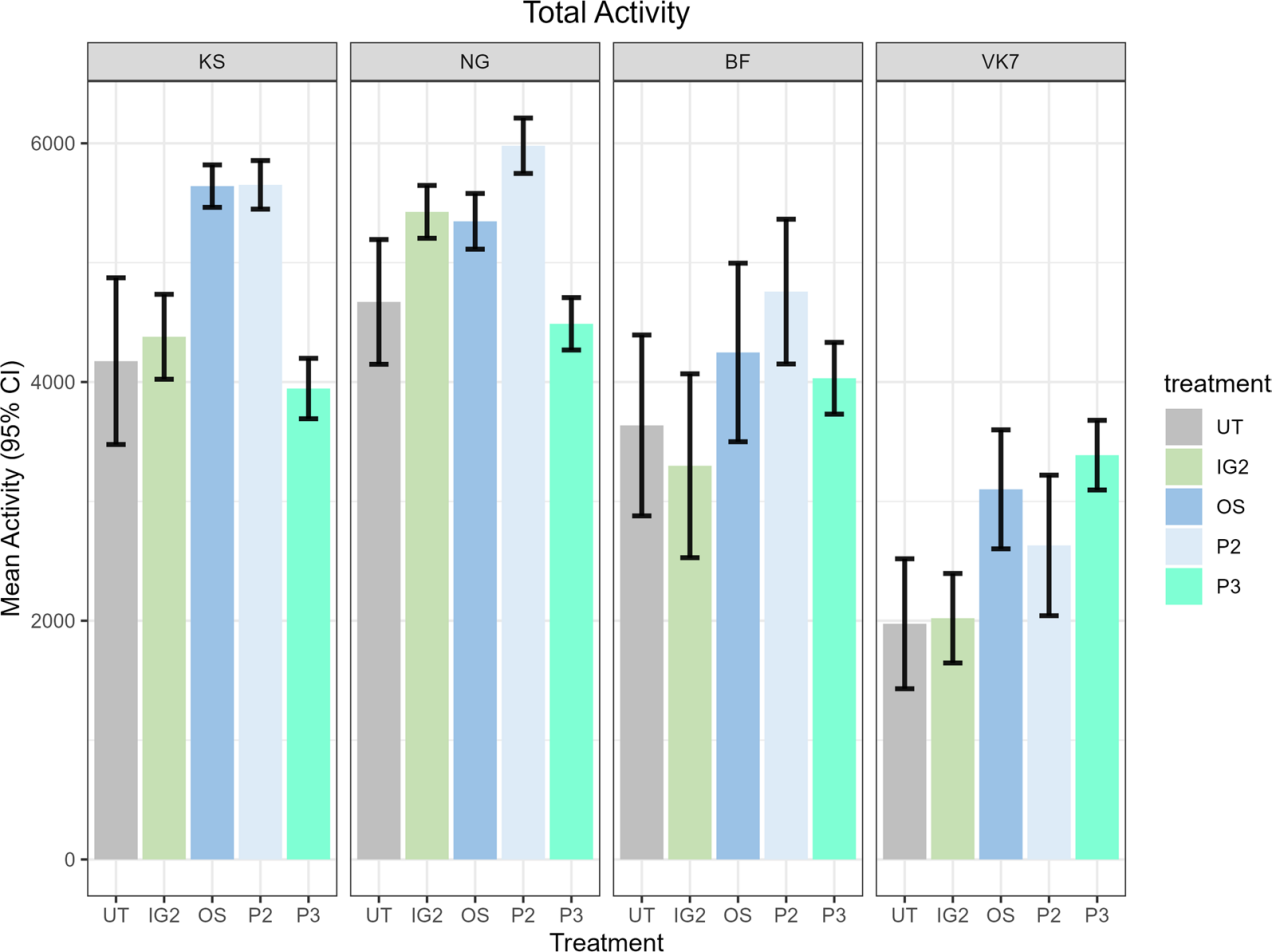
Total mosquito movement activity for all net treatments grouped by strain. Baseline behaviour on UT net is at the left side of each panel; 95% CI are indicated by error bars

A total of 3725 mosquitoes were assessed over 745 WHO cone tests using strains of KS, NG, BF and VK7 exposed to P2, OS, P3, IG2 and UT nets. Mosquitoes exposed to UT nets had a propensity to crawl along the net surface (Fig. 2a). The mean total movement events observed during cone tests on UT nets was 4175 (SD 2154) in KS, 4671 (SD 1655) in NG, 3636 (SD 1619) in BF and 1975 (SD 1769) in VK7 (Fig. 3). Susceptible strains typically had higher total activity than resistant strains. During exposure to treated nets, mosquito activity became dispersed throughout the cone and crawling on the net surface was reduced (Fig. 2b). The mean total movement events (Fig. 3) during ITN tests by strain was 4905 (SD 1120) in KS, 5326 (SD 904) in NG, 4022 (SD 1470) in BF, and 2737 (SD 1582) in VK7. Significant differences between the total movement comparing UT nets and ITNs by strain are indicated in Additional file 2: Table S1.

### Regional mosquito activity at ITNs

**Fig. 4:**
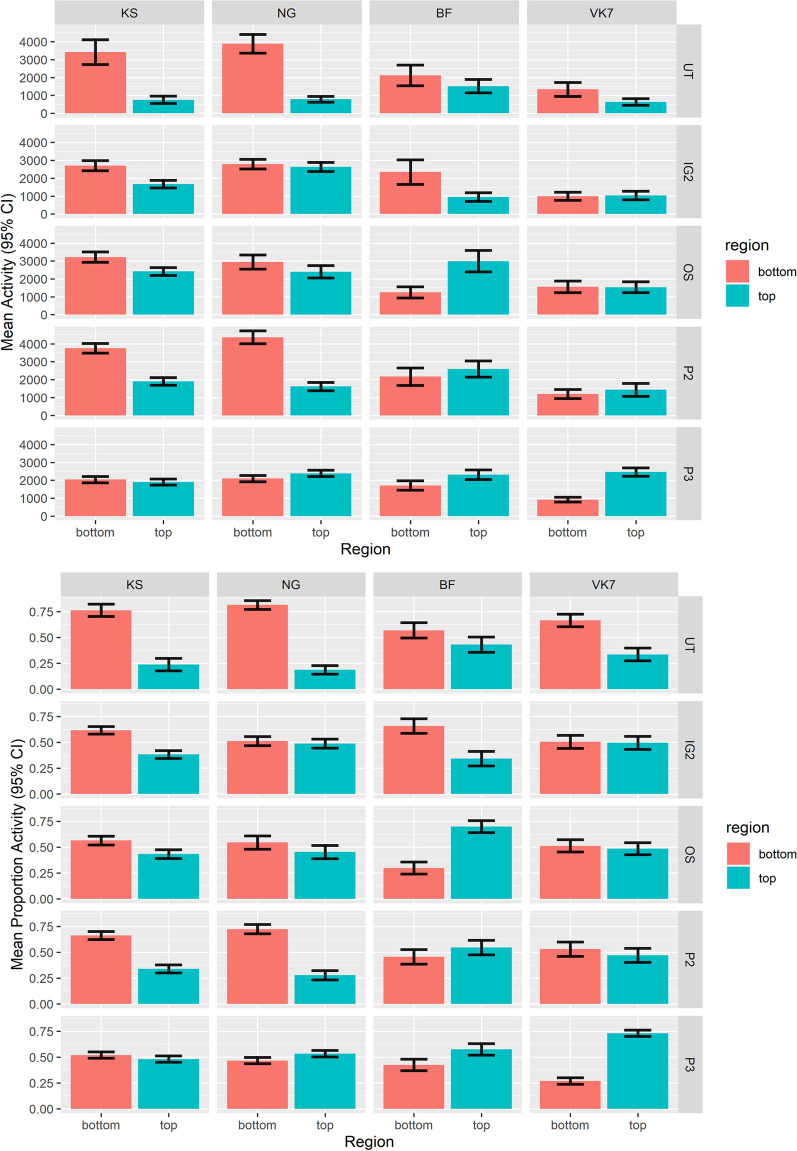
Regional activity in ViCTA analysis. **a** Mean absolute regional activity in LHC (‘bottom’) vs UHC (‘top’) regions for all strains and treatments. **b** Mean proportional regional activity. Baseline behaviour on UT net is shown in the top rows. 95% CI are indicated by error bars

The regional occupancy of mosquitoes in cones revealed variation in the responses of *Anopheles spp.* to different ITNs in terms of absolute (Fig. 4a) and relative (Fig. 4b) occupancy. During exposure to P2, mosquitoes were 1.48–2.11 times more active in the upper half of the cone (UHC) than in the UT net (Table 1, P2 vs UT upper region: KS OR 2.11; 95% CI 1.64, 2.7; *P* ≤0.0010; NG OR 1.78; 95% CI 1.39, 2.27; *P* ≤ 0.0010; BF OR 1.48; 95% CI 1.14, 1.92; *P* ≤ 0.0010; VK7 OR 1.67; 95% CI 1.29, 2.15, *P* ≤ 0.0010). Although activity was increased, there were no significant differences in the proportion of time spent in the upper and lower parts of the cone compared to the UT net (borderline result for KS Table 2 P2 vs UT: KS OR 0.51; 95% CI 0.25, 1.02; *P* = 0.0582).

**Table 1:** Region activity, insecticide-treated net versus untreated netting comparisons within *An. gambiae* strain from a generalised linear mixed model with a negative binomial distribution

Treatment contrast	Cone region	Strain	Incidence risk ratio, [95% CI], *P*-value
P2 vs UT	Lower	KS	0.77 [0.67, 0.89], < 0.0010*
P2 vs UT	Lower	NG	0.88 [0.77, 1.00], 0.0521
P2 vs UT	Lower	BF	0.79 [0.61, 1.01], 0.0583
P2 vs UT	Lower	VK7	0.67 [0.54, 0.84], < 0.0010*
P2 vs UT	Upper	KS	2.11 [1.64, 2.70], < 0.0010*
P2 vs UT	Upper	NG	1.78 [1.39, 2.27], < 0.0010*
P2 vs UT	Upper	BF	1.48 [1.14, 1.92], < 0.0010*
P2 vs UT	Upper	VK7	1.67 [1.29, 2.15], < 0.0010*
OS vs UT	Lower	KS	0.67 [0.58, 0.78], < 0.0010*
OS vs UT	Lower	NG	0.59 [0.51, 0.68], < 0.0010*
OS vs UT	Lower	BF	0.51 [0.36, 0.72], < 0.0010*
OS vs UT	Lower	VK7	0.76 [0.62, 0.93], 0.0036*
OS vs UT	Upper	KS	2.67 [2.10, 3.41], < 0.0010*
OS vs UT	Upper	NG	2.54 [2.01, 3.20], < 0.0010*
OS vs UT	Upper	BF	1.75 [1.31, 2.34], < 0.0010*
OS vs UT	Upper	VK7	1.55 [1.21, 1.98], < 0.0010*
IG2 vs UT	Lower	KS	0.73 [0.63, 0.85], < 0.0010*
IG2 vs UT	Lower	NG	0.61 [0.53, 0.70], < 0.0010*
IG2 vs UT	Lower	BF	1.29 [1.02, 1.62], 0.0271*
IG2 vs UT	Lower	VK7	0.68 [0.55, 0.84], < 0.0010*
IG2 vs UT	Upper	KS	2.2 [1.71, 2.83], < 0.0010*
IG2 vs UT	Upper	NG	3.12 [2.49, 3.90], < 0.0010*
IG2 vs UT	Upper	BF	0.72 [0.53, 0.98], 0.0327*
IG2 vs UT	Upper	VK7	1.41 [1.09, 1.82], 0.0034*
P3 vs UT	Lower	KS	0.68 [0.57, 0.80], < 0.0010*
P3 vs UT	Lower	NG	0.62 [0.53, 0.73], < 0.0010*
P3 vs UT	Lower	BF	0.83 [0.64, 1.07], 0.2299
P3 vs UT	Lower	VK7	0.45 [0.35, 0.56], < 0.0010*
P3 vs UT	Upper	KS	2.79 [2.16, 3.6], < 0.0010*
P3 vs UT	Upper	NG	3.38 [2.67, 4.27], < 0.0010*
P3 vs UT	Upper	BF	1.48 [1.13, 1.94], 0.0015*
P3 vs UT	Upper	VK7	2.43 [1.91, 3.08], < 0.0010*

Multiple pairwise comparisons, 95% confidence intervals and *P*-values corrected using the Dunnett adjustment

*UT* untreated net, *IG2* Interceptor^®^ G2 net, *OS* Olyset net, *P2* PermaNet 2.0 net, *P3* PermaNet 3.0 net, *KS* Kisumu, *NG* N’Gousso, *BF* Banfora

*Significant at 5% significance level

**Table 2:** Proportion of lower region activity, insecticide-treated net versus untreated netting comparisons within *An. gambiae* strain from a generalised linear mixed model with a beta-binomial distribution

Treatment contrast	Strain	Odds ratio, [95% CI], *P*-value
P2 vs UT	KS	0.51 [0.25, 1.02], 0.0582
P2 vs UT	NG	0.59 [0.29, 1.18], 0.1922
P2 vs UT	BF	0.56 [0.26, 1.22], 0.2117
P2 vs UT	VK7	0.72 [0.36, 1.42], 0.5579
OS vs UT	KS	0.34 [0.17, 0.67], < 0.001*
OS vs UT	NG	0.28 [0.14, 0.57], < 0.001*
OS vs UT	BF	0.25 [0.11, 0.59], < 0.001*
OS vs UT	VK7	0.60 [0.31, 1.19], 0.2062
IG2 vs UT	KS	0.50 [0.25, 0.99], 0.0470*
IG2 vs UT	NG	0.26 [0.13, 0.52], < 0.001*
IG2 vs UT	BF	1.74 [0.80, 3.75], 0.2372
IG2 vs UT	VK7	0.55 [0.28, 1.08], 0.1025
P3 vs UT	KS	0.35 [0.18, 0.71], < 0.001*
P3 vs UT	NG	0.23 [0.11, 0.46], < 0.001*
P3 vs UT	BF	0.55 [0.25, 1.21], 0.1930
P3 vs UT	VK7	0.23 [0.11, 0.45], < 0.001*

Multiple pairwise comparisons, 95% confidence intervals and *P*-values corrected using the Dunnett adjustment.

*UT *untreated net, *IG2* Interceptor^®^ G2 net, *OS* Olyset net, *P2* PermaNet 2.0 net, *P3* PermaNet 3.0 net, *KS* Kisumu, *NG* N’Gousso, *BF* Banfora

*Significant at 5% significance level

In OS tests, all strains spent 1.55–2.67 times more time in the UHC than in the UT net, and the proportional LHC occupancy was 66–75% lower (Table 1, OS vs UT upper region: KS OR 2.67; 95% CI 2.1, 3.41; *P* ≤ 0.0010; NG OR 2.54; 95% CI 2.01, 3.2; *P* ≤ 0.0010; BF OR 1.75; 95% CI 1.31, 2.34; *P* ≤ 0.0010; VK7 OR 1.55; 95% CI 1.21, 1.98, *P* ≤ 0.0010; Table 2 OS vs UT KS OR 0.34; 95% CI 0.25, 1.02; *P* = 0.0582; NG OR 0.59; 95% CI 0.29, 1.18; *P* = 0.1922; BF OR 0.56; 95% CI 0.26, 1.22; *P* = 0.2117; VK7 OR 0.72; 95% CI 0.36, 1.42, *P* = 0.5579); in P3 tests, mosquitoes spent 1.48–3.6 times more time in the UHC and were 65% less likely to move in the LHC (Table 1, P3 vs UT upper region: KS OR 2.79; 95% CI 2.16, 3.60; *P* ≤ 0.0010; NG OR 3.38; 95% CI 2.67, 4.27; *P* ≤ 0.0010; BF OR 1.48; 95% CI 1.13, 1.94; *P* = 0.0015; VK7 OR 2.43; 95% CI 1.91, 3.08, *P* ≤ 0.0010; Table 2, P3 vs UT: KS OR 0.35; 95% CI 0.18, 0.71; *P* ≤ 0.0010; NG OR 0.23; 95% CI 0.11, 0.46; *P* ≤ 0.0010; BF OR 0.55; 95% CI 0.25, 1.21; *P* = 0.1930; VK7 OR 0.23; 95% CI 0.11, 0.45, *P* ≤ 0.0010). Insecticide-susceptible and insecticide-resistant strains exhibited different behaviours during exposure to IG2: in the susceptible strains, between 50 and 74% less activity was observed in the LHC during IG2 tests compared to the UT net (Table 2 IG2 vs UT: KS OR 0.50; 95% CI 0.25, 0.99; *P* = 0.0470; NG OR 0.26; 95% CI 0.13, 0.52; *P* ≤ 0.0010), which was not observed in the two resistant strains (Table 2 IG2 vs UT: BF OR 1.74; 95% CI 0.8, 3.75; *P* = 0.2372; VK7 OR 0.55; 95% CI 0.28, 1.08; *P* = 0.1025).

### Resting behaviour

**Fig. 5:**
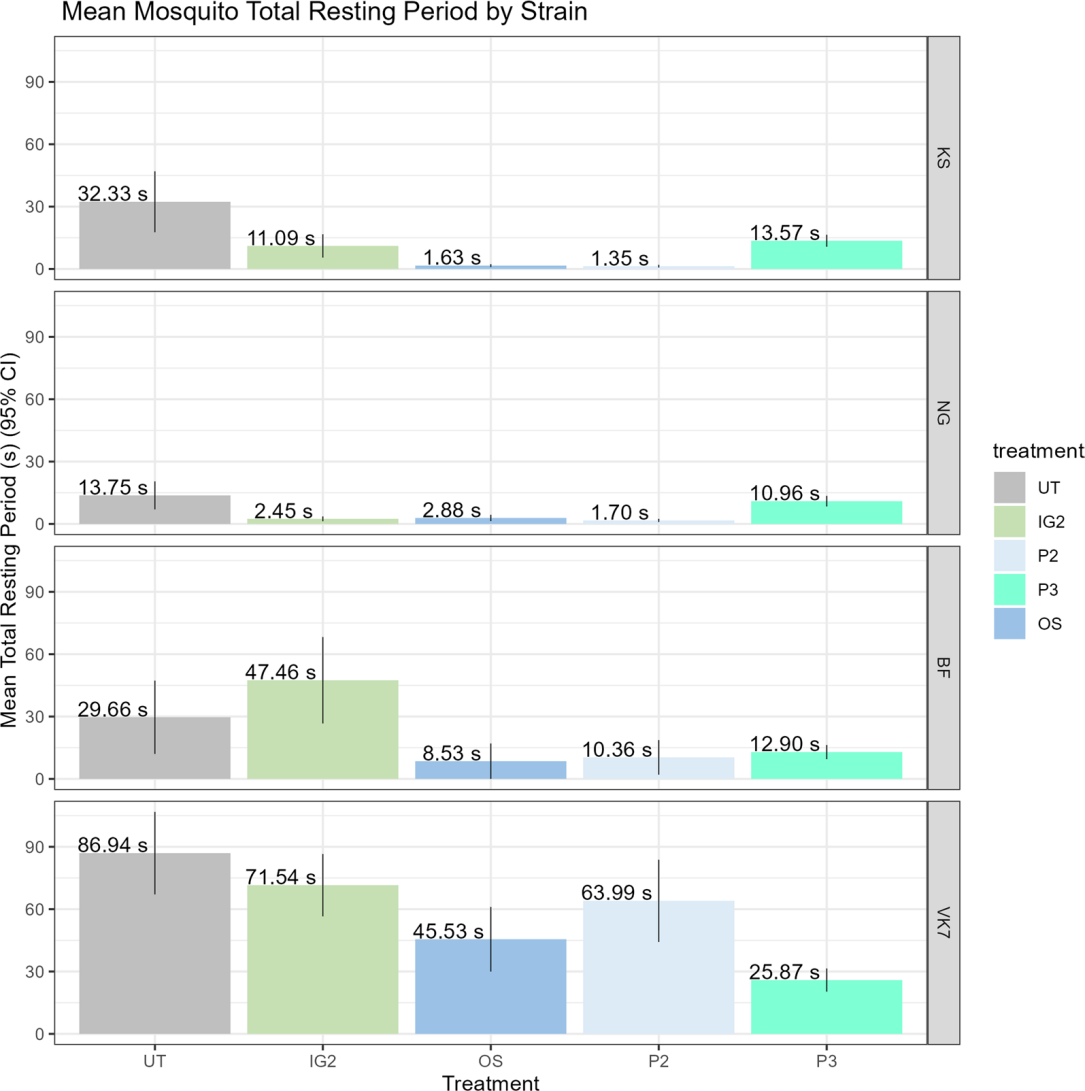
Mean mosquito resting period by strain and treatment. Seconds count based on number of measured frames where none of the five mosquitoes moved compared to the previous frame, out of a total of 1800 frames over the 3 min of the cone test. Numbers adjacent to each bar indicate mean number of seconds resting

Although mosquito resting could be indirectly inferred as the reciprocal of mosquito movement activity counts per frame (5 − *n*, where *n* is the number of moving mosquitoes detected per frame), a stricter measure was used. A resting frame was defined as a video frame where *none* of the five mosquitoes moved, compared to the previous frame. The total number of resting frames was calculated for each assay. Resting behaviour showed stark differences between susceptible and resistant strains (Fig. 5) with VK7 mosquitoes found to be resting for a large proportion of the cone assays.

### Blood-feeding success

Over 92% of mosquitoes successfully blood-fed after tests with UT nets, with most mosquitoes feeding at 1 h [KS 91.7% (176/192); NG 96% (192/200); BF 95.88% (93/97) and VK7 93.43% (199/213)]. After ITN tests, none of the mosquitoes exposed to P3, or the susceptible strains exposed to P2 and OS, survived long enough to feed. Of the few KS and NG mosquitoes that lived long enough to take a blood meal after IG2 tests, approximately half fed at 1 h PE (PE) [KS 53.85% (7/13), NG 53.33% (16/30)]. In the resistant strains, compared to UT nets, BF mosquitoes were at least 90% less likely to feed at 1 h PE when exposed to ITNs (Additional file 2: Table S2 BF: IG2 OR 0.10; 95% CI 0.00, 2.56; *P* = 0.2325; OS OR 0.01; 95% CI 0.00, 0.25; *P* = 0.0028; P2 OR 0.00; 95% CI 0.00, 0.02; *P* ≤ 0.0010). P2 had the largest immediate inhibitory effect [blood-feeding success = 9.8% (9/92)], followed by OS [48.57% (17/35)] and IG2 [61.86% (73/118)]. At 24 h PE, approximately one third of BF mosquitoes (31.11%, 14/45) that were unfed at 1 h after IG2 exposure, and all mosquitoes that were unfed at 1 h after exposure to P2 and OS tests, blood-fed (P2 *n* = 83; OS *n* = 18).

ITN exposure did not have a significant effect on the blood-feeding behaviour of VK7 mosquitoes at either 1 h or 24 h PE. Most mosquitoes successfully fed at 1 h [P2: 93.90% (77/82); OS: 87.50% (42/48); IG2 62.07% (18/29)].

### Blood meal weight

**Fig. 6:**
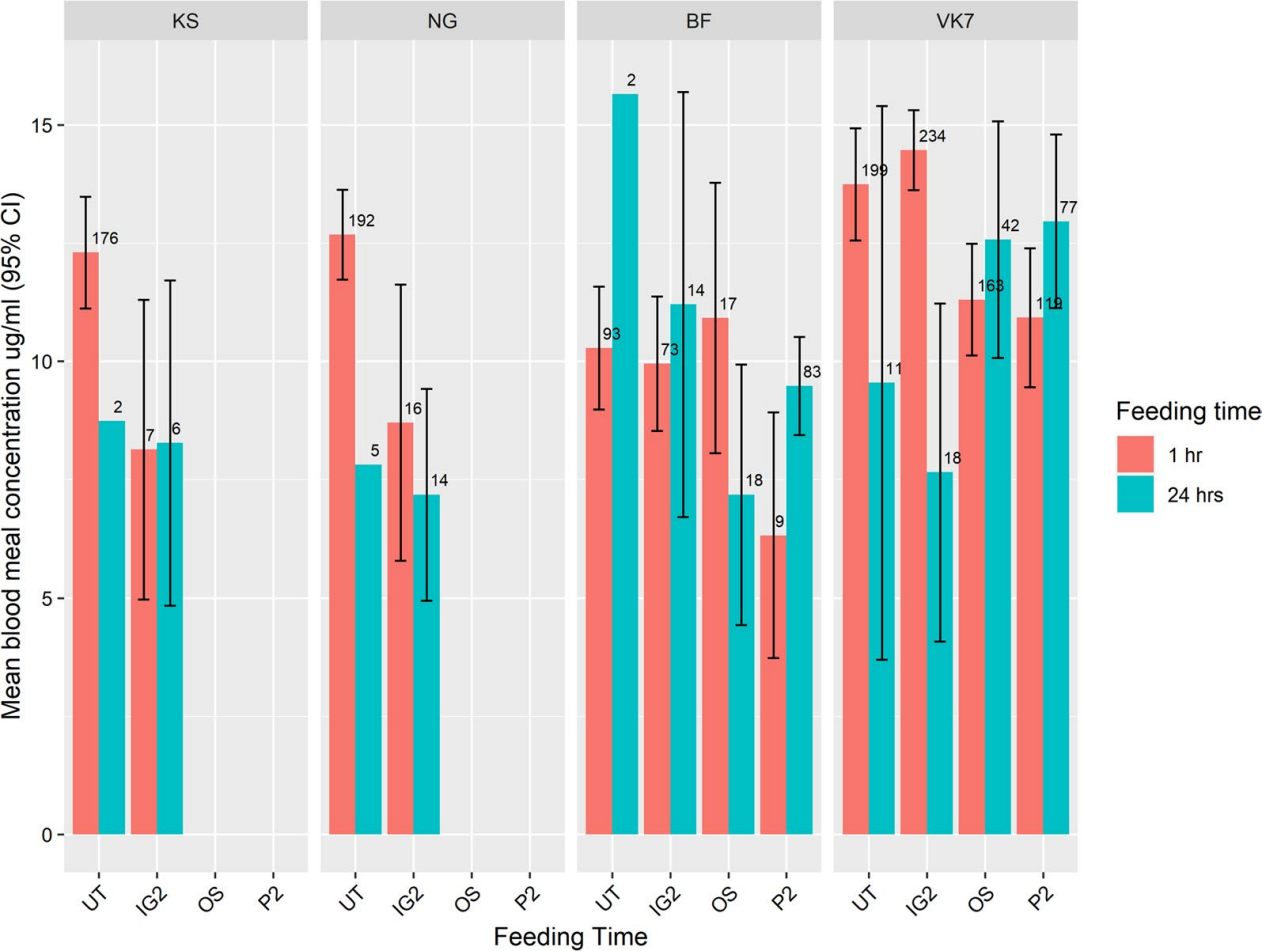
Mean haematin concentration of blood-fed mosquitoes which took a blood meal at 1 h and 24 h. Error bars at 95% CI are included only for cases where *n* > 2. Numbers adjacent to each bar indicate sample size

The mean total weight of blood meals taken after tests with UT netting was 12.47 µg (SD = 7.64). BMW per strain on UT netting was as follows: KS 12.26 µg (SD = 7.89), NG 11.74 µg (SD = 6.13), BF 10.48 µg (SD = 6.42) and VK7 13.10 µg (SD = 8.93). After ITN exposure, blood meal weights decreased (range 6.86 µg to 12.20 µg Fig. 6 and Additional file 2: Table S3), significantly so in NG and VK7 strains (NG *χ*^2^ = 5.47; *df* = 1; *P* = 0.0193; VK7 *χ*^2^ = 13.94; *df* = 3; *P* = 0.0030), where, compared to UT, at least 4.0 µg less blood was ingested (Additional file 2: Table S3 NG IG2 = −4.44 µg; 95% CI −8.23, −0.65; *P* = 0.0225, VK7: OS = −4.06 µg; 95% CI −6.84, −1.27; *P* = 0.0045 and P2 = −4.19 µg; 95% CI −7.12, −1.36; *P* = 0.0042). Activity in the LHC during tests was significantly associated with smaller blood meal weights in VK7 mosquitoes, and blood meals became 9.3 times smaller as activity increased (estimate = −9.35 µg; 95% CI −17.76, −0.93; *P* = 0.0298) regardless of treatment.

In VK7 mosquitoes that could not feed at 1 h PE but which recovered to feed at 24 h PE, blood meal weights were 1.45 times smaller blood meals compared to those that fed at 1 h PE (estimate = −1.45 µg; 95% CI −2.93, −0.03; *P* = 0.0551). The sizes of individual mosquitoes had a significant effect on blood meal weights in the NG, BF and VK7 strains (NG *χ*^2^ = 5.94; *df* = 1; *P* = 0.0148; BF *χ*^2^ = 17.55; *df* = 1; *P* < 0.0001; and VK7 *χ*^2^ = 11.18; *df* = 1; *P* = 0.0008) and as size increased, so did the size of the blood meal (NG estimate = 5.49; 95% CI 0.91, 10.03; *P* = 0.0201, BF estimate = 8.28; 95% CI 4.09, 12.47; *P* ≤ 0.0010 and VK7 estimate = 4.19; 95% CI 1.73, 6.65; *P* ≤ 0.0010).

### Longevity

After UT net tests, the 24-h mortality was 0.48–5.45% and the median longevity was 12–14 days (Additional file 2: Table S4, Additional file 2: S5). For all ITNs, 24-h mortality for KS and NG was 85.37–100% and 3.46–20.83% for resistant BF and VK7 (Additional file 2: Table S4). No mosquitoes survived P3 exposure and no susceptible mosquitoes survived P2. The median longevity for resistant strains after ITN tests was 9–14 days (Additional file 2: Table S5). KS and NG mosquitoes were at least 47.55 times more likely to die when exposed to IG2 or OS compared to a UT net (Additional file 2: Table S4).

The longevity of KS mosquitoes was influenced by activity during tests and mosquitoes were 11.93 times more likely to die as the LHC activity increased regardless of treatment (including UT). In VK7 mosquitoes, observed longevity also was influenced by the activity in the cone during tests (treatment*activity interaction: *χ*^2^ = 9.11; *df* = 3; *P* = 0.0278) and as the proportion of activity in the LHC increased, the likelihood of dying increased for UT and P2 (Additional file 2: Fig. S3a). In IG2 tests, mosquitoes were more likely to die when greater activity was recorded in the UHC, and, compared with UT, mosquitoes were significantly more likely to die when the proportion of activity close to the net was less than 50% (Additional file 2: Fig. S3b).

Mosquitoes that did not blood-feed at 1 h PE were at least 17 times more likely to die at 24 h PE (Additional file 2: Table S4 KS *χ*^2^ = 0.00; *df* = 1; *P* = 0.9987, NG *χ*^2^ = 25.80; *df* = 1; *P* < 0.0001, BF *χ*^2^ = 19.40; *df* = 1; *P* < 0.0001 and VK7 *χ*^*2*^ = 24.54; *df* = 1; *P* < 0.0001). The amount of blood ingested had a slight impact on longevity: BF and VK7 mosquitoes were 7% and 2% less likely to die as the BMW increased, respectively (Additional file 2: Table S5 BF HR 0.93; 95% CI 0.90, 0.93; *P* < 0.0001 and VK7 HR 0.98; 95% CI 0.97, 0.99; *P* = 0.0012). The effect of treatment on longevity varied with wing length for all strains, for example, smaller mosquitoes lived longer PE to UT netting (all strains) and were more likely to die if they were exposed to IG2 for all the strains except BF (Additional file 2: Fig. S2 a, b).

## Discussion

The laboratory methods that are currently used to evaluate ITN performance suffer from a lack of correlation between laboratory and semi-field findings, and the WHO cone test, the most widely implemented laboratory test, does not incorporate mosquito–host responses, mosquito–ITN interactions or post-exposure life history monitoring into its outcome measures [41]. Incorporating video capture and static scan-sampling analysis into the WHO cone test method in a previous study revealed differences in the behaviours of insecticide-susceptible and resistant mosquitoes in response to pyrethroid ITNs, which were modified depending on whether a human host attractant was present [14]. In this study the potentially subjective and time-consuming (approximately 10 min per video) manual scan sampling analysis was replaced by an automated movement quantification method that detected the mosquitoes’ spatial location and movements at 0.1 s intervals, providing an increase from 36 data sample points to 1800 in a typical run time of less than 2 min. In addition to confirming the irritant responses to ITNs previously shown [14, 42], in this study we demonstrate that the amount that a mosquito moves when exposed to an ITN directly impacts subsequent life history such as blood meal sizes and post-exposure life spans.

Within the cone, all mosquitoes responded to ITNs, reducing the amount of time spent crawling on the net surface. Mosquitoes responded the least strongly to PermaNet 2.0, confirming observations in previous wind tunnel experiments that showed a lack of close-range excito-repellent effects during exposure to deltamethrin-treated ITNs [27] and demonstrating that activity tracking in a small-scale cone can be used instead of additional experimental set-ups to characterise the excito-repellent and irritant properties of ITNs. Olyset Net and P3 nets provoked the strongest irritant responses; such responses to OS have been observed previously [42], whilst the increased irritant responses that were observed in PermaNet 3.0 compared to PermaNet 2.0 could be due to the doubled concentration of deltamethrin in the combination net [43, 44].

The amount and location of mosquito activity within the cone had a direct influence on the subsequent observed longevity. Kisumu strain mosquitoes were much more likely to die if they spent more time active at the net surface, even during UT net tests. Notably, in Interceptor G2 tests, the more time in which resistant mosquitoes spent flying away from the net surface, the higher the risk of dying. It has been previously found that the testing modality used for Interceptor G2 bioassays influences observed mortality [25, 45]. Our results suggest that mosquito activity during bioassays forms a key component of subsequent mortality, and that capturing such activity can be useful in understanding the impact of chemistries with delayed mortality effects.

All ITNs tested inhibited blood-feeding at 1 h PE in the resistant BF strain and in the few insecticide-susceptible mosquitoes that survived. By 24 h, the response to host cues was restored in mosquitoes exposed to P2 and OS nets. This waning of inhibition, combined with previous studies demonstrating that mosquitoes that feed successfully through a permethrin-treated net both recover from and lose permethrin-associated feeding inhibition [42, 46], suggests that the blood-feeding inhibition observed at 1 h would not be repeated if those mosquitoes were offered subsequent blood meals. As mosquitoes may encounter ITNs multiple times during their life span, incorporating multiple exposures and blood-feeding opportunities into post-test monitoring may enable this test system to record a more realistic ‘whole life’ impact on mosquitoes [24, 42, 46, 47]. Although no significant effects on blood-feeding success in VK7 mosquitoes were observed in post-ITN exposure, as the amount of movement in the lower half of the cone increased, the size of the blood meal ingested was reduced (regardless of whether the meal was at 1 or 24 h PE). This reduced blood meal weight had significant effects on longevity in resistant strains: BF mosquitoes were 7.1% less likely to die per unit of blood meal weight increase, and VK7 mosquitoes were 2.2% less likely to die per unit of blood meal weight increase.

The changes observed in the character of mosquito–ITN interactions within an overall lack of significant changes in total mosquito activity suggest that the observed irritancy with the four tested ITNs is unlikely to reduce mosquito contact time below that required to deliver a lethal dose of insecticide [48]. Such profiles could be used by bednet developers to assess the likely impact of new chemistries on mosquito behaviour and optimise the net profile for maximum mortality or sub-lethal impairment [42]. Furthermore, the ability to identify strain-specific differences, such as the different responses shown by insecticide-susceptible and insecticide-resistant mosquito strains to IG2 exposure, could be useful to both manufacturers who wish to design a bednet to target a specific mosquito population and to control programmes aimed at assessing the likely efficacy of an ITN against local vector populations.

As the ViCTA method uses an augmentation of the WHO cone assay, it has some limitations imposed by the assay design. The shape of the cone is not an ideal ‘window’ through which to observe flight behaviour; the curvature of the cone affects the light transmission, and focusing at the cone sides and the lip at the top of the cone can temporarily occlude moving mosquitoes. The placement angle of the camera (designed to capture the net surface in addition to the upper parts of the cone) and the steeply inward tapering of the cone impede the absolute determination of vertical mosquito flight, and thus changes in cone region occupancy are best evaluated as relative occupancy proportions. A more bespoke equipment setup such as that used in the baited box study [49] would resolve some of these issues, but an advantage of the ViCTA test is the use of a standard cone assay configuration. The background subtraction mechanism used in the ViCTA algorithm detects only *moving* mosquitoes, and the resting period (inactivity) of individual mosquitoes may only be inferred (as 5 − *n*, where *n* is the number of moving mosquitoes per frame). The stricter measure of resting used in this report (consecutive frames where *none* of the five mosquitoes moved) demonstrated large differences in resting between susceptible and resistant strains (Fig. 5) on UT and treated nets. It is interesting to speculate as to how the genetic changes which confer resistance to these strains may also impact mosquito behaviour, resulting in the relative lack of activity during the assays. A more complex time series analysis of resting behaviour and spatio-temporal patterns of activity during the course of the test is underway and may yet yield more subtle behavioural features.

## Conclusions

By using a simple augmentation of the WHO cone bioassay combined with automated video analysis we were able to demonstrate a wide range of complex behavioural responses of mosquitoes in response to insecticide-treated nets during the bioassay. Such behavioural responses (for example, the strong mosquito preference for the top surface of a bednet) have already been proven invaluable in the translation of this information to bednet designs [48, 50], with some dual-active-ingredient (AI) ITNs exploiting these preferences by specifically adding higher concentration or combination AIs to these regions [51]. Detailed behavioural insights can speed up the development process whilst reducing development costs and minimising the public health and financial risks of deploying ineffective vector control tools. Furthermore, these novel assays exploit complex video recording and information processing techniques that are now found in inexpensive and widely available technologies, helping to democratise their widespread adoption.

### Supplementary Information

**Additional file 1: Figure S1.** Flowchart of image analysis pipeline during a ViCTA experiment (top) and output files exported at the end of an experiment (bottom).

**Additional file 2: Figure S2.** Treatment effect on longevity as the wing length measurement increases by *An. gambiae* strain based on the fitted mixed-effects Cox regression model. **a** Effect by treatment, **b** contrast (insecticide-treated net [ITN] vs untreated netting) effect, *KS* Kisumu, *NG* N’Gousso, *BF* Banfora, *UT* untreated net, *IG2* Interceptor^®^ G2 net, *OS* Olyset net, *P2* PermaNet 2.0 net, and the solid black horizontal line is the reference (panel b, *HR* 1) with values above 1 showing more likelihood of dying when exposed to an ITN compared to UT and vice versa. **Figure S3**. Treatment effect on longevity as the activity at the lower half of the cone increases based on the fitted mixed-effects Cox regression model (VK7 strain only). **a** Effect by treatment, **b** contrast (insecticide-treated net [ITN] vs untreated netting) effect, *UT* untreated net, *IG2* Interceptor^®^ G2 net, *OS* Olyset net, *P2* PermaNet 2.0 net, and the solid black horizontal line is the reference (panel b, *HR* 1) with values above one showing more likelihood of dying when exposed to an ITN compared to UT and vice versa. **Table S1.** Total movement activity, insecticide treated net versus untreated netting comparisons within *An. gambiae* strain from a linear regression model. Multiple pairwise comparisons 95% confidence intervals and *P*-values corrected using the Dunnett adjustment. *UT* untreated net, *IG2* Interceptor^®^ G2 net, *OS* Olyset net, *P2* PermaNet 2.0 net, *P3* PermaNet 3.0 net, *KS* Kisumu, *NG* N’Gousso and *BF* Banfora. **Table S2.** Treatment comparisons for blood-feeding success post-exposure based on generalised linear mixed models (GLMMs) with a binomial distribution fitted for each *An. gambiae *strain separately within the time the blood meal was offered (1 or 24 h). Only the predictor variables in the final model for each strain are provided. *KS* Kisumu, *NG* N’Gousso, *BF* Banfora, *UT* untreated net, *IG2* Interceptor^®^ G2 net, *P2* PermaNet 2.0 net, *OS* Olyset net, *Ref* reference group, *OR* odds ratio, *CI* confidence interval, *significant at 5% significance level. Random effects include testing day and replicate. **Table S3.** Blood meal size results based on linear mixed effects models fitted for each *An. gambiae *strain separately. Only the predictor variables in the final model for each strain are provided. *KS* Kisumu, *NG* N’Gousso, *BF* Banfora, *UT* untreated net, *IG2* Interceptor^®^ G2 net, *P2* PermaNet 2.0 net, *OS* Olyset net, *Ref* reference group, *CI* confidence interval, *SD* standard deviation, cone activity = predicted proportion of activity at the lower half of the cone during exposure (behaviour data), *DE* during exposure, *significant at 5% significance level. Random effects include testing day and replicate. **Table S4.** Mortality at 24-h results based on generalised linear mixed models (GLMMs) with a binomial distribution fitted for each *An. gambiae *strain separately within the time the blood meal was offered (1 or 24 h). Only the predictor variables in the final model for each strain are provided. *KS* Kisumu, *NG* N’Gousso, *BF* Banfora, *UT* untreated net, *IG2* Interceptor^®^ G2 net, *P2* PermaNet 2.0 net, *OS* Olyset net, *Ref* reference group, *OR* odds ratio, *CI* confidence interval, *significant at 5% significance level. Random effects include testing day and replicate. **Table S5.** Longevity results based on the final model mixed-effects Cox regression models fitted for each *An. gambiae *strain separately. Only the predictor variables in the final model for each strain are provided. *KS* Kisumu, *NG* N’Gousso, *BF* Banfora, *UT* untreated net, *IG2* Interceptor^®^ G2 net, *P2* PermaNet 2.0 net, *OS* Olyset net, *Ref* reference group, *CI* confidence interval, *SD* standard deviation, *min* minimum, *max* maximum, *HR* hazard ratio, cone activity = predicted proportion of activity at the lower half of the cone during exposure (behaviour data), *DE* during exposure, *significant at 5% significance level and ^+^overall descriptive results. Random effects include testing day and replicate.

## Data Availability

ViCTA software and designs for cone test rig equipment are freely available on request to authors.
