# Supplementary material for: Video augmentation of the WHO cone assay to quantify mosquito behavioural responses to insecticide-treated nets
Source: Parasit Vectors. 2023 Nov 15;16:420. doi: 10.1186/s13071-023-06029-z (PMC10652617; doi:10.1186/s13071-023-06029-z)
Supplement: Supplementary file 1 — Additional file 1: Figure S1. Flowchart of image analysis pipeline during a ViCTA experiment (top) and output files exported at the end of an experiment (bottom). [file 13071_2023_6029_MOESM1_ESM.docx]

**This document contains additional supplementary information regarding the Video Cone Test Analysis (ViCTA) system.**

The software was written at LSTM Behaviour group, developed in Java using Netbeans development environment on a Windows 10 system. The software is freely available for use and can run on a standard Windows PC with a Java Virtual Machine installed.

An overview of the video analysis pipeline is shown in Fig. S1. The flowchart illustrates the iterative sequential processing of video frames from a cone test recording at 0.1s intervals. Image processing operations and background subtraction methods use the OpenCV software library (OpenCV version 2.5). The pipeline performs any required initial pre-processing on a greyscale transformed video frame (Gaussian smoothing filter and median noise filter). The pre-processed image is then passed to a Mixture of Gaussian background segmentation model. The background subtraction model analyses a series of sequential video frames in an attempt to classify moving (classed as foreground) versus non-moving (classed as background) regions. The output of this model is a binary image with a black background and white positions representing detected moving objects. This output is post-processed by morphological operators to remove isolated noise particles and to form solid areas representing candidate contours.

The candidate contours are assessed to ensure that they are located within the cone mask region and to ensure that their size is within the range of values corresponding to mosquito sizes (conforming to a distribution of mosquito sizes recorded during the software development process). Any contours outside the mask or the size ranges are removed from the candidate list. In the event of strong light reflections the candidate contours are also assessed by their brightness, with mosquitoes over a threshold removed from the candidate list. The number of contours per frame is recorded, along with the contour coordinates (X,Y of contour centroid) and size (contour area, in pixels) for each contour. Any frames with more than 5 contours present after post-processing are rejected from the analysis. The number of rejected frames is stored and used as a quality control metric (pValid, the proportion of valid frames within a recording). Rejected frames were typically caused by inadvertent movement of the experimental rig or sudden changes in lighting (generating a large number of false contours due to the sudden change in movement). In the initial stages of development the operators were given instructions on how to minimise movement of the equipment during the cone test recording and to minimise reflections on the cone from lighting in the room or movement within the room.

Composite images are recorded during the course of the experiment to indicate overall mosquito movement patterns. The *summary* composite image of mosquito movement is generated by creating an initial spatial image with the same width and height dimensions as the video frame with all pixels set to a maximal 8-bit brightness value of 255. At each frame the software incrementally adds to this image at regions where the minimum brightness in each current image frame is less than that of the corresponding pixel value in the composite image. The *detection* composite image is initialised as blank black colour image and is added to at each frame by writing red pixels corresponding to the positions of the centroids of detected movement in that frame. Finally, the *overlay* composite image is comprised of the actual mosquito positions shown in the *summary* image overlaid with the red detection pixels of the *detection* composite. Analysis of a single cone test video depends upon the base performance of the operating PC but typically takes less than two minutes.

Examples of composite images are shown in Fig. 2 of the main manuscript. The composite images are merely an indicator of overall behaviour and can also be used as a coarse checking method by the operator to assess overall performance of the method. A more detailed ‘per frame’ method was used to assess detection performance during development, whereby each video frame analysed was saved to disk for manual checking, with candidate contours overlaid on every frame by a crosshair pattern indicating the centroid of the detected candidate contour.

The software also outputs text log files including a *movement* text file containing positions of detected mosquitoes at every sampled frame, and a *results summary* file of experiment results in a format ready to paste into Microsoft Excel data entry sheet for further analysis. Inactive frames were calculated by running a Java script on the contents of the *movement* file to recover the number of inactive individual frames and to generate a total inactivity score and a score aggregating inactivity at 5s time intervals. Custom R scripts were used to extract the information from the data entry sheets into a format amenable for statistical analysis.


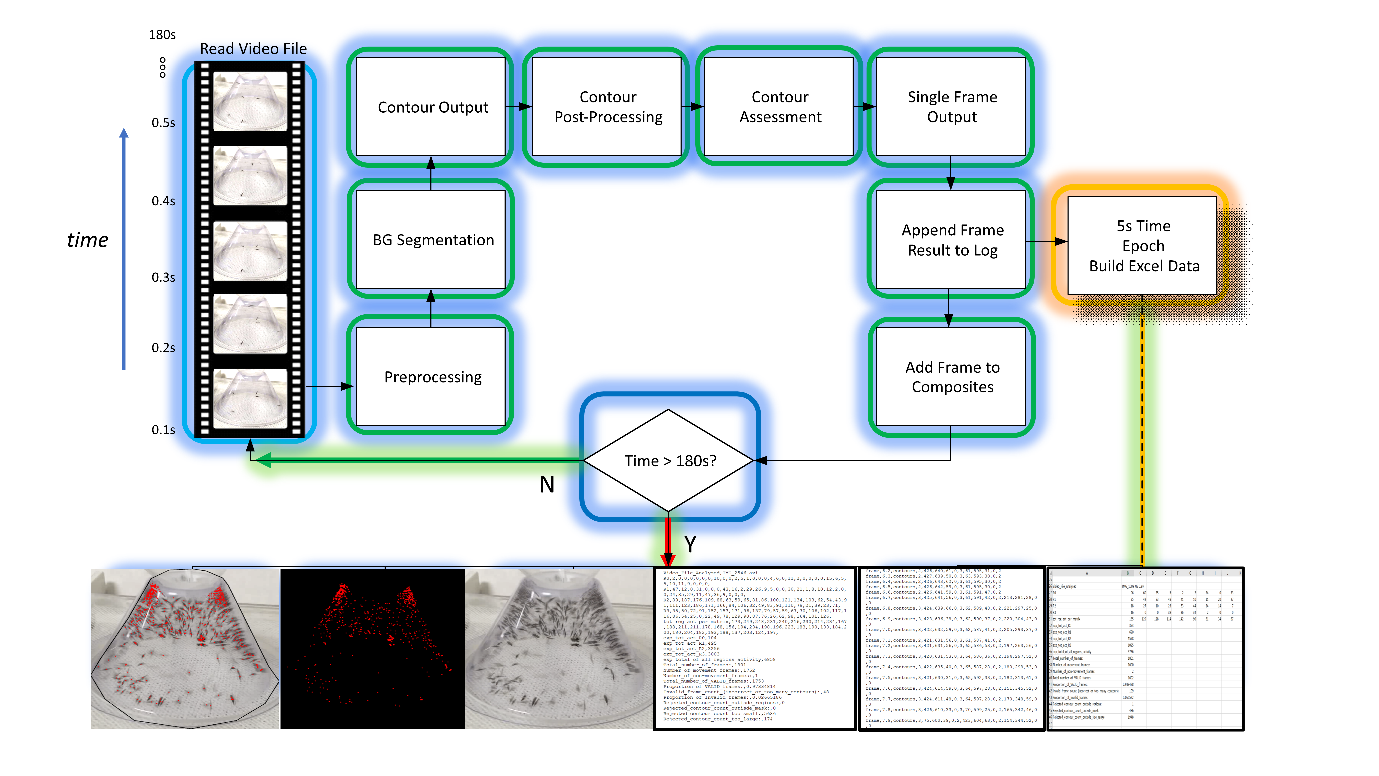


**Additional file 1: Figure S1.** Flowchart of image analysis pipeline during a ViCTA experiment (top) and output files exported at the end of an experiment (bottom).
