# Supplementary material for: Video augmentation of the WHO cone assay to quantify mosquito behavioural responses to insecticide-treated nets
Source: Parasit Vectors. 2023 Nov 15;16:420. doi: 10.1186/s13071-023-06029-z (PMC10652617; doi:10.1186/s13071-023-06029-z)
Supplement: Supplementary file 2 — Additional file 2: Figure S2. Treatment effect on longevity as the wing length measurement increases by An. gambiae strain based on the fitted mixed-effects Cox regression model. a Effect by treatment, b contrast (insecticide-treated net [ITN] vs untreated netting) effect, KS Kisumu, NG N’Gousso, BF Banfora, UT untreated net, IG2 Interceptor® G2 net, OS Olyset net, P2 PermaNet 2.0 net, and the solid black horizontal line is the reference (panel b, HR 1) with values above 1 showing more likelihood of dying when exposed to an ITN compared to UT and vice versa. Figure S3. Treatment effect on longevity as the activity at the lower half of the cone increases based on the fitted mixed-effects Cox regression model (VK7 strain only). a Effect by treatment, b contrast (insecticide-treated net [ITN] vs untreated netting) effect, UT untreated net, IG2 Interceptor® G2 net, OS Olyset net, P2 PermaNet 2.0 net, and the solid black horizontal line is the reference (panel b, HR 1) with values above one showing more likelihood of dying when exposed to an ITN compared to UT and vice versa. Table S1. Total movement activity, insecticide treated net versus untreated netting comparisons within An. gambiae strain from a linear regression model. Multiple pairwise comparisons 95% confidence intervals and P-values corrected using the Dunnett adjustment. UT untreated net, IG2 Interceptor® G2 net, OS Olyset net, P2 PermaNet 2.0 net, P3 PermaNet 3.0 net, KS Kisumu, NG N’Gousso and BF Banfora. Table S2. Treatment comparisons for blood-feeding success post-exposure based on generalised linear mixed models (GLMMs) with a binomial distribution fitted for each An. gambiae strain separately within the time the blood meal was offered (1 or 24 h). Only the predictor variables in the final model for each strain are provided. KS Kisumu, NG N’Gousso, BF Banfora, UT untreated net, IG2 Interceptor® G2 net, P2 PermaNet 2.0 net, OS Olyset net, Ref reference group, OR odds ratio, CI confidence interval, *signi [file 13071_2023_6029_MOESM2_ESM.docx]

ViCTA Manuscript Additional Figures and Tables V3

**Figures List**


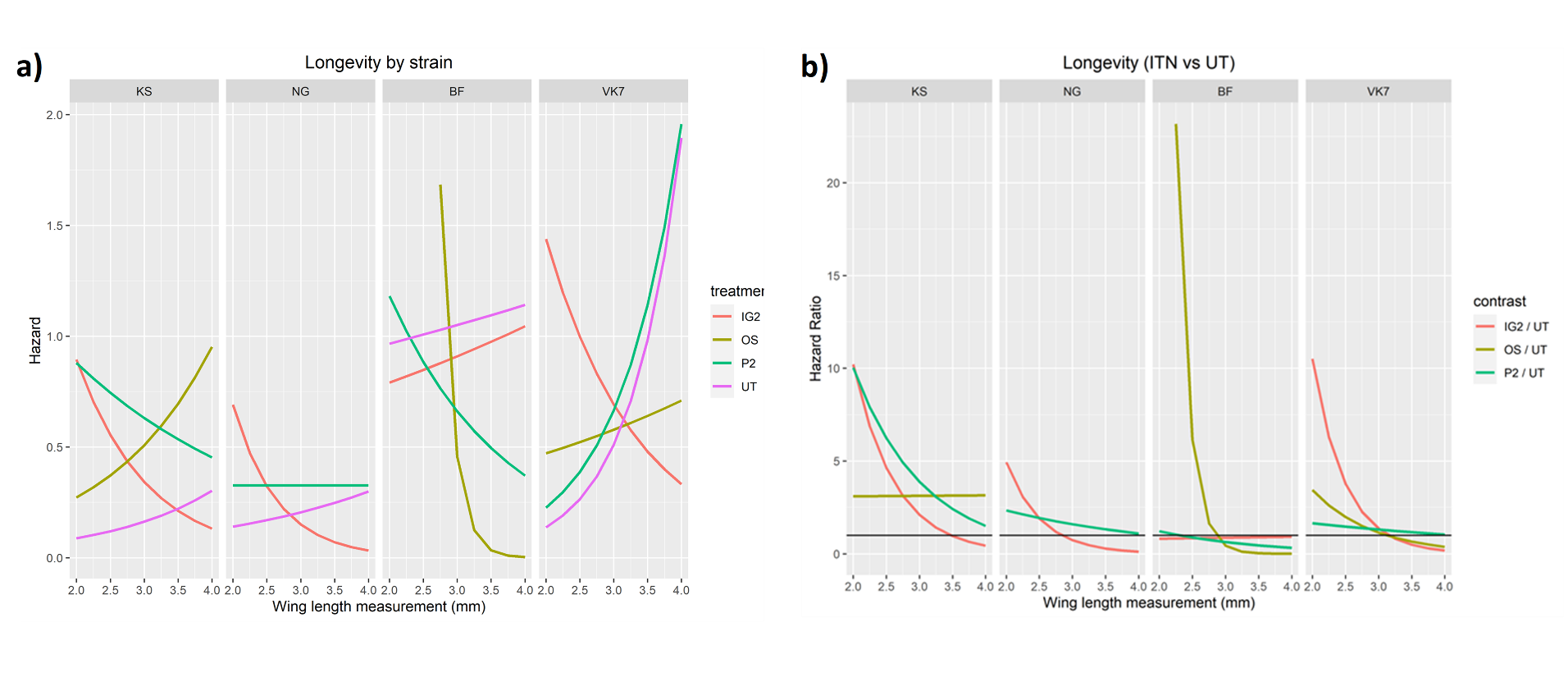


**Additional file 2: Figure S2:** Treatment effect on longevity as the wing length measurement increases by *An. gambiae* strain based on the fitted Mixed-effects Cox Regression Model. Where a) is the effect by treatment, b) the contrast (insecticide treated net (ITN) vs untreated netting) effect, KS = Kisumu, NG = N’Gousso, BF = Banfora, UT = Untreated net, IG2 = Interceptor® G2 net, OS = Olyset net, P2 = PermaNet 2.0 net, and the solid black horizontal line is the reference (panel b, HR = 1) with values above one showing more likelihood of dying when exposed to an ITN compared to UT and vice versa.


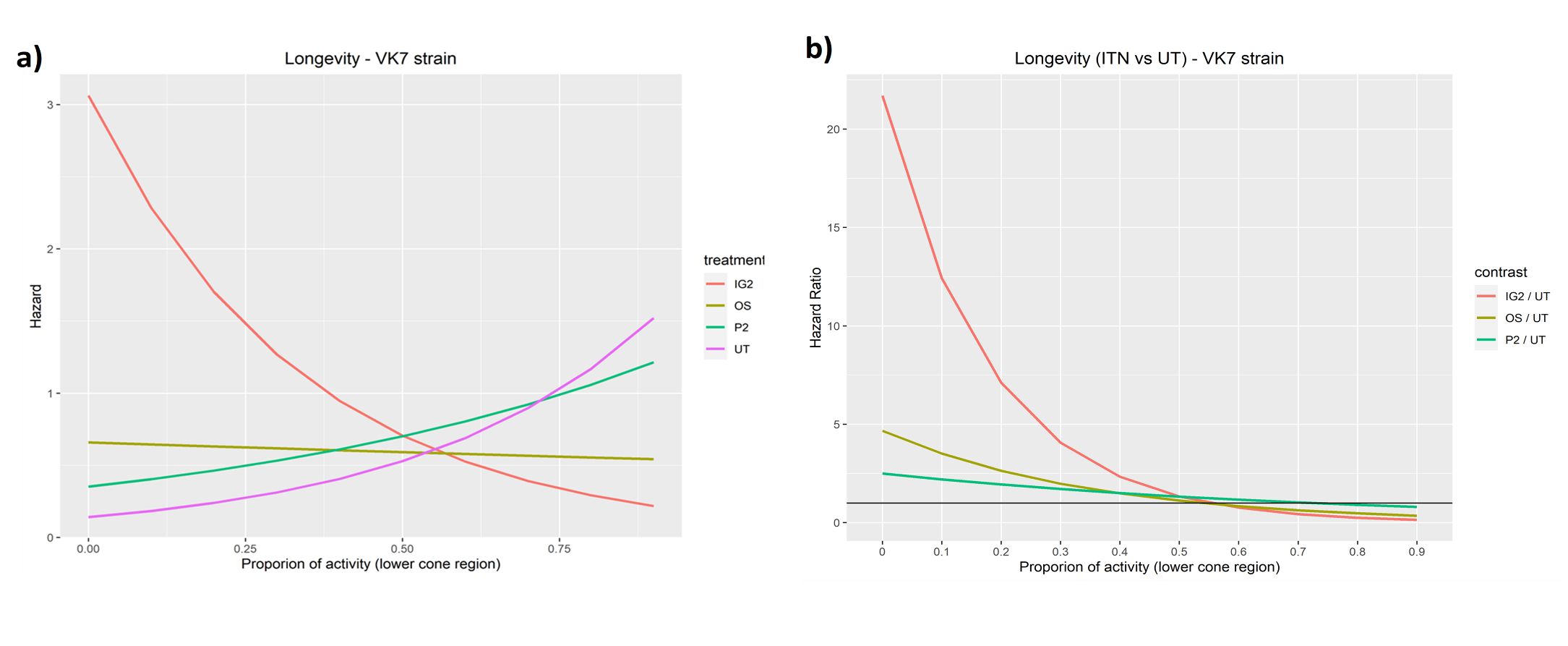


**Additional file 2: Figure S3**: Treatment effect on longevity as the activity at the lower half of the cone increases based on the fitted Mixed-effects Cox Regression Model (VK7 strain only). Where a) is the effect by treatment, b) the contrast (insecticide treated net (ITN) vs untreated netting) effect, UT = Untreated net, IG2 = Interceptor® G2 net, OS = Olyset net, P2 = PermaNet 2.0 net, and the solid black horizontal line is the reference (panel b, HR = 1) with values above one showing more likelihood of dying when exposed to an ITN compared to UT and vice versa.

**Tables List**

| **Treatment**  **Contrast** | **Strain** | **Mean Difference, [95% CI], P-value** |
| --- | --- | --- |
| P2 vs UT | KS | 1477.09 [768.48, 2185.69], <0.0010* |
| P2 vs UT | NG | 1308.14 [608.55, 2007.73], <0.0010* |
| P2 vs UT | BF | 1121.23 [125.62, 2116.84], 0.0211* |
| P2 vs UT | VK7 | 656.35 [-34.96, 1347.65], 0.0686 |
| OS vs UT | KS | 1466.33 [753.57, 2179.08], <0.0010* |
| OS vs UT | NG | 675.07 [-24.52, 1374.66], 0.0624 |
| OS vs UT | BF | 611.44 [-584.73, 1807.60], 0.5191 |
| OS vs UT | VK7 | 1126.27 [446.71, 1805.82], <0.0010* |
| IG2 vs UT | KS | 204.25 [-496.56, 905.06], 0.8437 |
| IG2 vs UT | NG | 754.36 [88.83, 1419.90], 0.0201* |
| IG2 vs UT | BF | -337.88 [-1293.85, 618.09], 0.7626 |
| IG2 vs UT | VK7 | 46.52 [-604.77, 697.81], 0.9942 |
| P3 vs UT | KS | -229.52 [ -946.61, 487.57], 0.8080 |
| P3 vs UT | NG | -183.52 [ -891.70, 524.65], 0.8800 |
| P3 vs UT | BF | 395.20 [ -612.48, 1402.88], 0.7059 |
| P3 vs UT | VK7 | 1412.68 [717.13, 2108.24], <0.0010* |

**Additional file 2: Table S1. Total movement activity**, insecticide treated net versus untreated netting comparisons within *An. gambiae* strain from a Linear Regression Model. Multiple pairwise comparisons 95% Confidence Intervals and P-values corrected using the Dunnet adjustment. Where UT = Untreated net, IG2 = Interceptor® G2 net, OS = Olyset net, P2 = PermaNet 2.0 net, P3 = PermaNet 3.0 net, KS = Kisumu, NG = N’Gousso and BF = Banfora.

|  | | | **Frequency/n (%)** | | | | **OR [95% CI], P-value** | | |
| --- | --- | --- | --- | --- | --- | --- | --- | --- | --- |
| **strain** | **Feeding time (hrs)** | **Fed** | **UT** | **IG2** | **OS** | **P2** | **IG2 vs UT** | **OS vs UT** | **P2 vs UT** |
| KS | 1 | Yes | 176/192 (91.67) | 7/13 (53.85) | - | - | 0.02 [0.00; 0.48], 0.0175* | - | - |
|  |  | No | 16/192 (8.33) | 6/13 (46.15) | - | - |  |  |  |
|  | 24 | Yes | 2/16 (12.50) | 6/6 (100.00) | - | - | - | - | - |
|  |  | No | 14/16 (87.50) |  | - | - |  |  |  |
| NG | 1 | Yes | 192/200 (96.00) | 16/30 (53.33) | - | - | 0.01 [0.00; 0.17], 0.0011* | - | - |
|  |  | No | 8/200 (4.00) | 14/30 (46.67) | - | - |  |  |  |
|  | 24 | Yes | 5/8 (62.50) | 14/14 (100.00) | - | - | - | - | - |
|  |  | No | 3/8 (37.50) | - | - | - |  |  |  |
| BF | 1 | Yes | 93/97 (95.88) | 73/118 (61.86) | 17/35 (48.57) | 9/92 (9.78) | 0.10 [0.00; 2.56], 0.2325 | 0.01 [0.00; 0.25], 0.0028* | 0.00 [0.00; 0.02], <0.0010* |
|  |  | No | 4/97 (4.12) | 45/118 (38.14) | 18/35 (51.43) | 83/92 (90.22) |  |  |  |
|  | 24 | Yes | 2/4 (50.00) | 14/45 (31.11) | 18/18 (100) | 83/83(100) | 0.40 [0.01; 22.14], 0.8793 | - | - |
|  |  | No | 2/4 (50.00) | 31/45 (68.89) | - | - |  |  |  |
| VK7 | 1 | Yes | 199/213 (93.43) | 234/263 (88.97) | 163/211 (77.25) | 119/201 (59.20) | 0.53 [0.03; 9.68], 0.8879 | 0.16 [0.01; 2.90], 0.3148 | 0.06 [0.00; 1.03], 0.0527 |
|  |  | No | 14/213 (6.57) | 29/263 (11.03) | 48/211 (22.75) | 82/201 (40.80) |  |  |  |
|  | 24 | Yes | 11/14 (78.57) | 18/29 (62.07) | 42/48 (87.50) | 77/82 (93.90) | 0.28 [0.01; 8.93], 0.6914 | 0.56 [0.02; 18.46], 0.9371 | 1.36 [0.04; 45.64], 0.9835 |
|  |  | No | 3/14 (21.43) | 11/29 (37.93) | 6/48 (12.50) | 5/82 (6.10) |  |  |  |

**Additional file 2: Table S2. Treatment comparisons for blood feeding success post-exposure** based on generalised linear mixed effects models (GLMMs) with a binomial distribution fitted for each *An. gambiae* strain separately within the time the blood meal was offered (1 or 24 hours). Only the predictor variables in the final model for each strain provided. Where KS = Kisumu, NG = N’Gousso, BF = Banfora, UT = Untreated net, IG2 = Interceptor® G2 net, P2 = PermaNet 2.0 net, OS = Olyset net, Ref = Reference group, OR = Odds ratio, CI = Confidence Interval and * = Significant at 5% significance level. Random effects include testing day and replicate.

| **Strain** | **Variable** | **N; Mean (SD)** | **Estimate [95% CI], P-value** |
| --- | --- | --- | --- |
| KS | Treatment: UT (Ref) | 170; 12.26 (7.89) | - |
|  | Treatment: IG2 | 13; 8.20 (3.21) | -2.81 [-7.99; 2.36], 0.2847 |
| NG | Treatment: UT (Ref) | 121; 11.74 (6.13) | - |
|  | Treatment: IG2 | 18; 7.72 (5.09) | -4.44 [-8.23; -0.65], 0.0225* |
|  | Wingspan | - | 5.47 [0.91; 10.03], 0.0201* |
| BF | Treatment: UT (Ref) | 78; 10.48 (6.42) | - |
|  | Treatment: IG2 | 75; 10.12 (6.33) | -0.02 [-2.5; 2.45], 0.9852 |
|  | Treatment: OS | 22; 6.86 (5.00) | -2.16 [-6.35; 2.04], 0.2942 |
|  | Treatment: P2 | 45; 9.82 (4.52) | 0.93 [-2.02; 3.88], 0.5248 |
|  | Wingspan | - | 8.28 [4.09; 12.47], <0.0010* |
| VK7 | Treatment: UT (Ref) | 132; 13.10 (8.93) | - |
|  | Treatment: IG2 | 153; 11.85 (7.10) | -1.8 [-4.07; 0.47], 0.1189 |
|  | Treatment: OS | 137; 11.93 (7.83) | -4.06 [-6.84; -1.27], 0.0045* |
|  | Treatment: P2 | 146; 12.20 (8.19) | -4.24 [-7.12; -1.36], 0.0042* |
|  | Wingspan | - | 4.19 [1.73; 6.65], <0.0010* |
|  | Feeding Time: 1h (Ref) | 444; 12.56 (7.83) | - |
|  | Feeding Time: 24h | 117; 11.81 (8.33) | -1.45 [-2.93; 0.03], 0.0551 |
|  | Feeding Time: Unfed | 7; 0.00 (0.00) | -8.58 [-13.67; -3.49], <0.0010* |
|  | Cone activity DE (proportion lower half) | - | -9.35 [-17.76; -0.93], 0.0298* |

**Additional file 2: Table S3. Blood meal size results** based on linear mixed effects models fitted for each *An. gambiae* strain separately. Only the predictor variables in the final model for each strain provided. Where KS = Kisumu, NG = N’Gousso, BF = Banfora, UT = Untreated net, IG2 = Interceptor® G2 net, P2 = PermaNet 2.0 net, OS = Olyset net, Ref = Reference group, CI = Confidence Interval, SD = Standard deviation, Cone activity = Predicted proportion of activity at the lower half of the cone during exposure (behaviour data), DE = During exposure and * = Significant at 5% significance level. Random effects include testing day and replicate.

| **Strain** | **Variable** | **Frequency 24h mortality/n (%)** | **OR [95% CI], P-value** |
| --- | --- | --- | --- |
| KS | Treatment: UT (Ref) | 3/191 (1.57) | - |
|  | Treatment: IG2 | 198/218 (90.83) | 1011921.8 [348.16; 2941169593.7], <0.0010* |
|  | Treatment: OS | 175/205 (85.37) | 317167.29 [16.27; 6183439064.05], 0.0120* |
|  | Fed at 1h: Yes (Ref) | 0/179 (100.00) | - |
|  | Fed at 1h: No | 376/435 (86.44) | 57228182585.28 [0.00; ∞], 0.9988 |
| NG | Treatment: UT (Ref) | 11/202 (5.45) | - |
|  | Treatment: IG2 | 227/260 (87.31) | 47.55 [5.84; 387.08], <0.0010* |
|  | Fed at 1h: Yes (Ref) | 8/205 (3.90) | - |
|  | Fed at 1h: No | 440/467 (94.22) | 75.39 [14.22; 399.73], <0.0010* |
| BF | Treatment: UT (Ref) | 4/98 (4.08) | - |
|  | Treatment: IG2 | 13/113 (11.50) | 1.15 [0.23; 5.85], 0.8653 |
|  | Treatment: OS | 17/54 (31.48) | 2.6 [0.44; 15.38], 0.2927 |
|  | Treatment: P2 | 20/96 (20.83) | 0.82 [0.15; 4.47], 0.8164 |
|  | Fed at 1h: Yes (Ref) | 5/185 (2.70) | - |
|  | Fed at 1h: No | 49/179 (27.84) | 17.76 [4.94; 63.89], <0.0010* |
| VK7 | Treatment: UT (Ref) | 1/207 (0.48) | - |
|  | Treatment: IG2 | 9/260 (3.46) | 5.36 [0.43; 66.28], 0.1906 |
|  | Treatment: OS | 19/218 (8.72) | 13.96 [0.68; 286.1], 0.0872 |
|  | Treatment: P2 | 9/209 (4.31) | 3.33 [0.14; 78.2], 0.4544 |
|  | Fed at 1h: Yes (Ref) | 10/691 (1.45) | - |
|  | Fed at 1h: No | 28/203 (13.79) | 39.81 [9.27; 171.02], <0.0010* |

**Additional file 2: Table S4. Mortality at 24-hrs results** based on generalised linear mixed effects models (GLMMs) with a binomial distribution fitted for each *An. gambiae* strain separately within the time the blood meal was offered (1 or 24 hours). Only the predictor variables in the final model for each strain provided. Where KS = Kisumu, NG = N’Gousso, BF = Banfora, UT = Untreated net, IG2 = Interceptor® G2 net, P2 = PermaNet 2.0 net, OS = Olyset net, Ref = Reference group, OR = Odds ratio, CI = Confidence Interval and * = Significant at 5% significance level. Random effects include testing day and replicate.

| **Strain** | **Effect** | Variable level | **N; Median [95% CI]** | **Mean (SD)** | **min-max** | **HR [95% CI], P-value** |
| --- | --- | --- | --- | --- | --- | --- |
| KS | Treatment and wingspan interaction: wing length mean = 3.00mm | Treatment: UT (Ref) | 130; 14 [12; 15] | 12.86 (7.54) | 0 - 31 | - |
|  |  | Treatment: IG2 | 161; 0 [0; 0] | 1.12 (3.52) | 0 - 17 | 2.12 [0.77; 5.85], 0.2017 |
|  |  | Treatment: OS | 164; 0 [0; 0] | 0.12 (0.32) | 0 - 1 | 3.12 [1.03; 9.48], 0.0426 * |
|  |  | Treatment: P2 | 180; 0 [0; 0] | 0.00 (0.00) | 0 - 0 | 3.89 [1.33; 11.44], 0.0083 * |
|  | Feeding time | Feeding time: 1h (Ref) | 127; 14 [13; 15] | 13.35 (7.31) | 1 - 31 | - |
|  |  | Feeding time: 24h | 6; 9 [3; 15] | 8.83 (4.75) | 3 - 15 | 1.68 [0.54; 5.21], 0.3696 |
|  |  | Feeding time: Unfed | 8; 6 [2; 13] | 6.88 (4.22) | 2 - 13 | 3.82 [1.80; 8.11], 0.0005 * |
|  |  | Feeding time: Other | 647; 0 [0; 0] | 0.11 (0.96) | 0 - 17 | 11.85 [5.08; 27.67], <0.0001 * |
|  | Cone activity DE | Proportion (lower half) | 788; 0 [0; 0] ^+^ | 2.36 (5.80) ^+^ | 0 - 31 ^+^ | 11.79 [3.40; 40.85], 0.0001 * |
| NG | Treatment and wingspan interaction: wing length mean = 2.94mm | Treatment: UT (Ref) | 139; 12 [10; 14] | 11.11 (6.40) | 0 - 31 | - |
|  |  | Treatment: IG2 | 188; 0 [0; 0] | 1.18 (3.78) | 0 - 24 | 0.83 [0.45; 1.52], 0.7794 |
|  |  | Treatment: OS | 172; 0 [0; 0] | 0.00 (0.00) | 0 - 0 | 1.63 [0.85; 3.16], 0.1952 |
|  |  | Treatment: P2 | 180; 0 [0; 0] | 0.00 (0.00) | 0 - 0 | 1.63 [0.84; 3.17], 0.1990 |
|  | Feed time | Feeding time: 1h (Ref) | 140; 12 [11; 14] | 11.49 (6.30) | 0 - 31 | - |
|  |  | Feeding time: 24h | 15; 7 [3; 12] | 7.60 (5.51) | 1 - 18 | 1.55 [0.84; 2.84], 0.1584 |
|  |  | Feeding time: Unfed | 3; 7 [-∞; ∞] | 7.00 (0.00) | 7 - 7 | 2.19 [0.68; 7.09], 0.1913 |
|  |  | Feeding time: Other | 681; 0 [0; 0] | 0.03 (0.66) | 0 - 17 | 21.47 [12.09; 38.12], <0.0001 * |
| BF | Treatment and wingspan interaction: wing length mean = 2.75mm | Treatment: UT (Ref) | 78; 13 [11; 14] | 13.13 (6.43) | 2 - 30 | - |
|  |  | Treatment: IG2 | 75; 12 [11; 15] | 13.92 (7.63) | 3 - 36 | 0.85 [0.51; 1.43], 0.7839 |
|  |  | Treatment: OS | 22; 10 [5; 11] | 9.10 (4.30) | 3 - 16 | 1.65 [0.73; 3.70], 0.3323 |
|  |  | Treatment: P2 | 45; 14 [13; 17] | 14.95 (5.35) | 5 - 26 | 0.74 [0.37; 1.48], 0.6040 |
|  | Blood meal size | Haematin weight value | 220; 13 [12; 14] ^+^ | 13.38 (6.66) ^+^ | 2 – 36 ^+^ | 0.93 [0.90; 0.96], <0.0001 * |
| VK7 | Treatment and wingspan interaction: wing length mean = 3.08mm ^+^ | Treatment: UT (Ref) | 132; 13 [10; 14] | 12.56 (6.40) | 2 - 26 | - |
|  |  | Treatment: IG2 | 153; 10 [8; 12] | 11.16 (6.60) | 2 - 28 | 1.14 [0.72; 1.81], 0.8105 |
|  |  | Treatment: OS | 137; 9 [9; 10] | 9.55 (4.26) | 3 - 27 | 1.03 [0.60; 1.77], 0.9920 |
|  |  | Treatment: P2 | 146; 10 [9; 12] | 11.19 (5.19) | 3 - 23 | 1.28 [0.70; 2.35], 0.6398 |
|  | Treatment and cone activity during exposure interaction: mean proportion of activity (cone lower half) = 0.53 ^+^ | Treatment: UT (Ref) | 132; 13 [10; 14] | 12.56 (6.40) | 2 - 26 | - |
|  |  | Treatment: IG2 | 153; 10 [8; 12] | 11.16 (6.60) | 2 - 28 | 1.14 [0.72; 1.81], 0.8105 |
|  |  | Treatment: OS | 137; 9 [9; 10] | 9.55 (4.26) | 3 - 27 | 1.03 [0.60; 1.77], 0.9920 |
|  |  | Treatment: P2 | 146; 10 [9; 12] | 11.19 (5.19) | 3 - 23 | 1.28 [0.70; 2.35], 0.6398 |
|  | Feeding time | Feeding Time: 1h (Ref) | 444; 10 [10; 11] | 11.12 (5.77) | 2 - 28 | - |
|  |  | Feeding Time: 24h | 117; 10 [9; 12] | 10.98 (5.62) | 2 - 27 | 0.87 [0.69; 1.10], 0.2409 |
|  |  | Feeding Time: Unfed | 7; 13 [2; 25] | 11.57 (9.48) | 2 - 25 | 0.38 [0.17; 0.85], 0.0196 * |
|  | Blood meal size | Haematin weight value | 568; 10 [10; 11] ^+^ | 11.10 (5.78) ^+^ | 2 – 28 ^+^ | 0.98 [0.97; 0.99], 0.0012 * |

**Additional file 2: Table S5. Longevity results** based on the final model mixed-effects Cox Regression Models fitted for each *An. gambiae* strain separately. Only the predictor variables in the final model for each strain provided. Where KS = Kisumu, NG = N’Gousso, BF = Banfora, UT = Untreated net, IG2 = Interceptor® G2 net, P2 = PermaNet 2.0 net, OS = Olyset net, Ref = Reference group, CI = Confidence Interval, SD = Standard deviation, min = minimum, max = maximum, HR = Hazard Ratio, Cone activity = predicted proportion of activity at the lower half of the cone during exposure (behaviour data), DE = During exposure, * = Significant at 5% significance level and ^+^ = Overall descriptive results. Random effects include testing day and replicate.
